# Metabolic and Hormonal Profile of Adolescent Girls With Polycystic Ovary Syndrome With Concomitant Autoimmune Thyroiditis

**DOI:** 10.3389/fendo.2021.708910

**Published:** 2021-07-02

**Authors:** Karolina Joanna Skrzyńska, Agnieszka Zachurzok, Aneta Monika Gawlik

**Affiliations:** Department of Pediatrics and Pediatric Endocrinology, School of Medicine in Katowice, Medical University of Silesia, Katowice, Poland

**Keywords:** polycystic ovary syndrome, cardiovascular disease, adolescent girls, autoimmune thyroiditis, metabolic disorder

## Abstract

**Introduction:**

Both polycystic ovary syndrome (PCOS) and autoimmune thyroiditis (AT) are considered to be among the most common endocrinopathies in young women, and they are classified as diseases that affect many processes in the human body. Their role in the development of metabolic disorders and diseases of the cardiovascular system in adult women is also emphasized. However, there are no data available to assess such risk in the teenage girl population. The aim of the study was to assess the hormonal and metabolic profile of adolescent girls with PCOS, additionally diagnosed with AT, as well as to identify possible risk factors for the coexistence of AT and PCOS.

**Material and Methods:**

80 euthyroidic PCOS patients were qualified for the study (chronological age 16.54 ± 1.00 years, BMI 24.60 ± 4.16 kg/m^2^). Eighteen girls diagnosed with AT were included in the study group and 62 girls without AT—in the control group. Each patient had biochemical and hormonal tests performed. Additionally, to diagnose AT, the level of antibodies against thyroid peroxidase (anti-TPO) and anti-thyroglobulin (anti-TG), as well as the image of the thyroid gland on ultrasound examination, were taken into account.

**Results:**

Estradiol concentration was significantly higher in the study than in the control group (203.00 ± 217.00 vs. 152.00 ± 78.50 pmol/L, p=0.02). Higher DHEAS concentrations were also observed in the AT group compared with the group without AT (391.28 ± 176.40 vs. 317.93 ± 114.27 µg/dl, p=0.04). Moreover, there was a positive correlation between AT and estradiol concentration (r_y_=0.27; p=0.04). It was also shown that there is a tendency toward statistical significance for the positive correlation between the positive anti-TPO titer and the glucose concentration at 120 min OGTT (r_ƴ_=0.26; p=0.07) and girls with PCOS and AT had higher glucose levels in 120 min OGTT (115.29±41.70 vs. 98.56±28.02 mg/dl, p=0.08).

**Conclusion:**

The study results showed no difference in the metabolic profile between the groups. The high concentration of estradiol found in girls with PCOS and AT may indicate the role of this hormone in the development of the autoimmune process. However, the numbers are small, and more research is needed to confirm our findings.

## Introduction

Polycystic ovary syndrome (PCOS) is claimed to be one of the most common endocrine diseases and according to some studies it might affect 6% to 15% of women of reproductive age (1) and 1% to 6% of teenage girls. This disease affects many processes in the human body and often has long-term health consequences, such as an increased risk of metabolic disorders and cardiovascular diseases (2).

Autoimmune thyroid disease is another group of widespread endocrine disorders—dominated by Hashimoto’s disease (HT) (3). As many as 5% to 20% of young women might suffer from this disease (4). Although many genetic and environmental triggers were identified, their connections and interactions are still unclear (5). The adverse influence of hypothyroidism on lipid metabolism disorders is stated (6). It is worth mentioning that the most important independent risk factor for cardiovascular diseases in children is the duration of thyroid dysfunction. The risk of cardiovascular disease seems to be higher if hypothyroidism or subclinical hypothyroidism is associated with autoimmune thyroiditis (AT). Recent studies indicate that the diagnosis of AT itself, without any comorbid disorders of thyroid gland, increases the risk of cardiovascular diseases (7). In adult PCOS women with AT the increase cardiovascular disease risk was identified (8). However, there are no data about such relationship in adolescent girls.

The aim of the study was to assess the hormonal and metabolic profile of adolescent girls with PCOS, additionally diagnosed with AT, as well as to identify possible risk factors for the coexistence of AT and PCOS.

## Materials AND METHODS

The study was retrospective and was completed on the basis of the collected hospital medical records of patients aged 12.33 to 18.00 years (median age, 16.54 ± 1.00 years) with newly diagnosed PCOS. The study included data from a total of 80 euthyroidic girls with PCOS. Eighteen girls—additionally diagnosed with AT were enrolled into the study group, while 62 PCOS subjects—without AT coexistence appeared in the control group. Seven patients (8.75%) from study group and 9 (14.06%) from control group had levothyroxine treatment introduced before diagnostic management of PCOS due to hypothyroidism.

The exclusion criteria were as follows: eating disorders (anorexia nervosa, bulimia), hyperprolactinemia (prolactin [PRL] ≥721 mIU/L), adrenal disorders (17hydroxyprogesterone [17OHP] ≥10 ng/ml, in patients with 17OHP between 2.0 and 9.9 ng/ml urine steroid profile results suggestive of congenital adrenal hyperplasia), abnormal thyroid stimulating hormone (TSH) level (TSH <0.4 mIU/L or >10.0 mIU/L), use of medications known to influence sex steroids in last 3 months. In all patients, the following data were analyzed:

- anthropometric measurements (body weight [kg], height [cm], BMI [kg/m^2^], gynecological age [years], age of menarche [years], hirsutism assessed according to the Ferriman-Gallwey scale [points]);
- biochemical tests results (total cholesterol — TC [mg/dl], triglycerides — TG [mg/dl], HDL cholesterol — HDL [mg/dl], LDL cholesterol — LDL [mg/dl], fasting glucose, and at 120 min of oral glucose tolerance test (OGTT) after a load of 75 g glucose [mg/dl]);
- hormonal tests results (total testosterone — T [ng/dl], estradiol — E2 [pmol/L], luteinizing hormone — LH [mIU/ml], follicle stimulating hormone — FSH [mIU/ml], dehydroepiandrosterone sulfate — DHEAS [µg/dl], 17OHP [ng/ml], androstenedione - A [ng/ml], fasting insulin, and 120 min OGTT [µIU/ml]), parameters of thyroid function (TSH [µIU/ml], free thyroxine — fT4 [ng/dl]),
- thyroid antibody (antiperoxydase antibody — anti-TPO [IU/ml], antithyreoglobulin antibody — anti-TG [IU/ml]).
- thyroid ultrasound — performed with 12 MHz linear transducer (Siemens Medical Solution USA, Inc). Heterogeneous, hypoechoic echotexture of the thyroid gland was considered to be characteristic for AT.
- HOMA IR (assessment of a model of homeostasis), FIGR (fasting insulin to glucose ratio), and LH to FSH ratio (LH/FSH) were calculated.

The diagnosis of PCOS was made according to the recommendations of consensus by Ibanez et al. (9). Menstrual disorders, present more than 2 years after menarche, were defined as oligomenorrhea (periods less frequent than every 45 days), polymenorrhea (cycles shorter than every 21 days), and secondary amenorrhea (no menstrual bleeding in the last 90 days). Clinical hyperandrogenism was considered to be hirsutism rated ≥8 points on the basis of the Ferriman-Gallwey scale. Biochemical hyperandrogenism was defined on the basis of total testosterone concentration >55 ng/dl (9).

The diagnosis of AT was made on the basis of positive anti-TPO (> 35 IU/ml) and/or anti-TG (>40 IU/ml) with the abnormal ultrasound scan of the thyroid gland (10). What is more, thyroid function abnormalities were not required for the diagnosis of AT.

Measurements of the concentrations of the tested hormones were made in the blood serum using the electrochemiluminescence method on the Cobas e411 apparatus (T, E2, TSH, fT4), chemiluminescence on the Immulite 2000XPi apparatus (LH, FSH, DHEAS, anti-TPO, anti-TG) or the ELISA method on the DS2 analyzer (17OHP, A).

Anthropometric data, biochemical, hormonal, and immunological results were compared using the Statistica 12 PL software. All values were expressed as mean ± standard deviation for normal or median (interquartile range) for skewed distribution. Comparison between groups was performed using Student *t* test for normally distributed data and Mann-Whitney U test for non-normally distributed samples. Correlation analysis was performed using Pearson correlation coefficient for normally distributed, and Spearman correlation coefficient for non-normally distributed data. Gamma correlation was used for non-normal distributions with many tied ranks. To evaluate variables effecting AT occurrence, positive anti-TPO, and anti-TG in PCOS girls, standard multiple regression analysis was performed with β coefficient interpretation with regard to the strength and direction of the relationship between variables. P value <0.05 was considered statistically significant, and 0.05<p ≤ 0.1 was considered as a trend toward statistical significance.

The Bioethics Committee of the Medical University of Silesia approved the study (KNW/0022/KB/70/16).

## Results

Clinical characteristics of the girls with PCOS with and without AT are shown in **Table 1**. The groups did not vary significantly in terms of chronological age, age of menarche or BMI (p>0.05). In the AT group, more severe hirsutism was observed and a shorter menstruation cycle length; however, the differences was statistically insignificant (p> 0.05).

**Table 1:** Clinical characteristics of patients with PCOS: with AT and without AT.

Variables	PCOS (n = 80)		p
	with AT (n = 18)	without AT (n = 62)	
Chronological age [years]	16.38 ± 1.14	16.16 ± 1.30	0.56
Age of menarche [years]	12.33 ± 0.97	12.30 ± 1.53	0.93
Average cycle length [days]	75.00 ± 52.50	104.00 ± 66.00	0.35
Hirsutism [points]	9.00 ± 3.50	3.50 ± 5.50	0.19
BMI [kg/m2]	23.21 ± 2.19	25.05 ± 4.49	0.29

AT, autoimmune thyroiditis; BMI, body mass index; PCOS, polycystic ovary syndrome.

In the study group, TSH levels had a tendency to be higher than in the control group (2.30 ± 1.07 µIU/ml vs. 1.65 ± 1.18 µIU/ml, p = 0.055), whereas fT4 concentration was similar in both groups (1.37 ± 0.26 ng/dl vs. 1.30 ± 0.16 ng/dl, p>0.05) The groups showed very similar parameters of lipid metabolism, which statistically did not vary considerably. Also, fasting glucose and insulin levels in both groups were similar and statistically did not vary significantly (p>0.05). However, glucose concentration at 120 min of OGTT was higher in the group of patients with AT and the difference tended to be statistically important (p = 0.08). The HOMA IR and FIGR ratios showed no differences between the groups (p > 0.05) (**Table 2**).

**Table 2:** Comparison of biochemical parameters in the subgroups of PCOS patients: with AT vs without AT.

Variable	PCOS (n = 80)		p
	with AT (n = 18)	without AT (n = 62)	
Glucose 0 [mg/dl]	88.67 ± 9.80	87.83 ± 6.95	0.69
Glucose 120 [mg/dl]	115.29 ± 41.70	98.56 ± 28.02	0.08
Insulin 0 [µIU/ml]	16.18 ± 6.68	15.16 ± 10.04	0.71
Insulin 120 [µIU/ml]	63.10 ± 62.20	55.10 ± 36.45	0.83
HOMA IR	3.61 ± 1.64	3.34 ± 2.26	0.65
FIGR	0.18 ± 0.07	0.17 ± 0.11	0.80
Total cholesterol [mg/dl]	165.35 ± 21.61	168.08 ± 30.33	0.73
HDL cholesterol [mg/dl]	53.95 ± 8.03	53.19 ± 10.73	0.79
LDL cholesterol [mg/dl]	93.01 ± 21.31	94.09 ± 24.98	0.87
Triglycerides [mg/dl]	82.00 ± 13.00	95.00 ± 23.00	0.35

AT, autoimmune thyroiditis; Glucose 0, fasting glucose; Glucose 120, glucose 120 minutes OGTT; FIGR, fasting insulin to glucose ratio; HOMA IR, assessment of a model of homeostasis; insulin 0, fasting insulin; insulin 120, insulin 120 minutes OGTT; PCOS, polycystic ovary syndrome.

The concentration of LH, FSH, and LH/FSH showed no significant differences between the groups. E2 concentration was significantly higher in the AT group (p = 0.02). Higher DHEAS concentrations were also observed in the AT group compared with the group without AT (p=0.04). Both T and 17OHP concentrations were slightly higher in the AT group but did not differ significantly between the groups (p>0.05) (**Table 3**).

**Table 3:** Hormonal characteristic of PCOS patients: with AT and without AT.

Variable	PCOS (n = 80)		p
	with AT (n = 18)	without AT (n = 62)	
LH [mIU/ml]	8.94 ± 5.17	8.36 ± 6.55	0.73
FSH [mIU/ml]	4.73 ± 1.39	4.84 ± 1.51	0.11
LH/FSH	1.66 ± 0.61	1.61 ± 0.74	0.91
Estradiol [pmol/l]	203.00 ± 217.00	152.00 ± 78.50	0.02
Testosteron [ng/dl]	64.28 ± 22.44	59.67 ± 19.55	0.40
DHEAS [µg/dl]	391.28 ± 176.40	317.93 ± 114.27	0.04
17OHP [ng/ml]	2.76 ± 1.15	2.57 ± 0.56	0.67
Androstendione [ng/ml]	4.98 ± 1.86	4.41 ± 1.28	0.50

17OHP, 17-hydroxyprogesterone; AT, autoimmune thyroiditis; DHEAS, dehydroepiandrosterone sulfate; FSH, follicle stimulating hormone; LH, luteinizing hormone; PCOS, polycystic ovary syndrome.

Moreover, we found a statistically significant correlation between AT and the concentration of E2 (r_y_=0.27; p=0.04) in patients in the study group. However, no significant correlation was found between AT and other hormone concentrations, including LH, FSH, LH/FSH, T, DHEAS, 17OHP, A. A statistically significant correlation was reported between the positive anti-TG and T concentration (r_ƴ_=0.34; p=0.04), DHEAS (r_ƴ_=0.42; p=0.01), 17OHP (r_ƴ_=0.37; p=0.03), and A (r_ƴ_=0.55; p=0.001). Yet, no significant correlations were found between the concentrations of LH, FSH, LH/FSH, and E2 and the presence of positive anti-T.

In the study group, a significant tendency was stated for the positive correlation between AT diagnosis and glucose concentration at 120 min of OGTT (r_ƴ_=0.26; p=0.07). Apart from that, no statistically significant correlations between AT and other parameters of carbohydrate metabolism were observed. Neither TSH nor fT4 showed any correlation with BMI in the described group.

In multiple regression analysis, age, BMI, LH/FSH, estradiol, testosterone, DHEAS were included in the analysis as scale variables. The estradiol and DHEAS were significantly associated with AT occurrence (β=0.28, p=0.03, β=0.27, p=0.05, respectively) and positive anti-TPO (β=0.28, p=0.03, β=0.30, p=0.03, respectively). Anti-TG was related significantly only with DHEAS concentration (β=0.32, p=0.02).

## Discussion

The study aims to assess the hormonal and metabolic profile of adolescent girls with PCOS, additionally diagnosed with AT, as well as attempt to identify a group of PCOS patients with the highest risk of AT. Eighty PCOS patients were enrolled in the study, among them — 18 girls diagnosed with AT were included in the study group and the others 62 girls in the control group. The estradiol concentration was significantly higher in the AT group than in the control group (p=0.02). Higher DHEAS concentrations were also observed in the AT group compared to the group without AT (p=0.04). There was also reported a tendency toward statistical significance for the positive correlation between the positive anti-TPO titer and the glucose concentration at 120 min OGTT and girls both with PCOS and AT had higher glucose levels in 120 min OGTT (p=0.08). Moreover, there was a correlation between AT and estrogen concentration (r_y_=0.27; p=0.04).

Many studies on PCOS and disorders of the thyroid gland (including AT) indicate an increased risk of metabolic disorders and cardiovascular diseases in this group of patients (11–13). The risk seems to be higher if hypothyroidism or subclinical hypothyroidism co-exist with AT. Recent studies indicate that the diagnosis of AT itself, without any comorbid disorders of thyroid gland increases the risk of cardiovascular diseases (7). On the other hand, referring to the thyroid disorders themselves — without AT, the latest data indicates that the risk of cardiovascular complications is definitely the highest in patients not treated for hypothyroidism but also treated improperly, i.e., keeping TSH levels during treatment in the lower range or above the normal level.

Disorders of lipid and carbohydrate metabolism including insulin resistance and arterial hypertension (14–16) are reported to be the most common causes leading to cardiological diseases in women with PCOS. There are also other risk factors observed in these women such as oxidative stress, blood clotting disorders, impaired endothelial function, increased arterial stiffness, increased inflammatory markers, or dysfunction of the heart muscle. In patients with PCOS and AT, Ho et al. (8) proved an increased risk of diabetes, lipid metabolism disorders, and ischemic heart disease compared with the control group (AT without PCOS). However, an increased risk of strokes in the study group was not stated. On the other hand, Kim et al. (17) observed significantly higher BMI, hip circumference, fasting insulin and glucose levels, and HOMA IR in their PCOS and AT patients compared with patients with PCOS but without AT. Worth mentioning is that any lipid metabolism disorder did not differ significantly between the described groups. In our study, we also did not observe an increased risk of lipid disorders in the group of adolescent girls with PCOS and AT. Only glucose concentration in 120 min OGTT was higher in this group and tended to be statistically significant. It cannot be excluded that if cohort size would be bigger or the age of studied patients older the differences would reach statistical significance. Abnormal glucose concentration at 120 min OGTT is supposed to be a risk factor for type 2 diabetes, so it can be suspected that the coexistence of PCOS and AT might be associated with a higher risk of carbohydrate metabolism disorders (18). The other test results did not differ significantly between the groups. In addition, there are no studies in the literature assessing metabolic disorders in the population of adolescent girls with PCOS and AT. What is more, among adult women, there is not much information on these issues at the same time. However, it seems that the coexistence of these two disease entities may increase the risk of metabolic disorders or cardiovascular diseases as demonstrated by Ho et al. (19). It should be considered that the described metabolic disorders may be, above all, secondary to excessive body weight. On the other hand, a systematic review of the literature and a meta-analysis reported a lower concentration of adiponectin in women with PCOS after excluding the influence of BMI as a confounding factor. Furthermore, reduced adiponectin concentration is associated with an increased risk of insulin resistance (20). In our group of girls with PCOS and AT, despite a higher glucose concentration at 120 min of OGTT, BMI was slightly lower than in girls with PCOS without AT; however, the difference did not reach statistical significance. Thus, the occurrence of metabolic complications in women with PCOS may be independent of excess body weight (20). Despite the lack of unambiguous data, it seems that every patient with PCOS and AT, regardless of age or weight, ought to be monitored for the possible occurrence of carbohydrate metabolism disorders. Also, the increasing frequency of hypothyroidism with the duration of AT may additionally increase the cardiovascular risk in this group of patients.

Both epidemiological data showing the expansion of thyroid diseases in women and reported in the literature the more frequent occurrence of thyroid diseases, especially AT, in women with PCOS may suggest a potential influence of sex hormones on the pathomechanism of the coexistence of these two diseases. Sex hormones (estrogens, progesterone, and testosterone) have an impact on the functioning of the immune system. Additionally, in women with PCOS an increased ratio of estradiol to progesterone due to long oligo- or anovulatory periods and luteal insufficiency might be involved in the stimulation of the immune system and consequently in the development of AT. During the menstrual cycles, the increased concentration of estradiol in the follicular phase leads to a conversion of the immune response from Th1-dependent to Th2-dependent and consequently to the production of Th2-induced cytokines (21). Additionally, estradiol activates FOXP3 (forkhead box P3, skurfin), a transcription factor that plays a role in the regulation of the immune response, which is responsible for the production of the T regulatory cells (Tregs). Estradiol also increases the activity of B lymphocytes and stimulates the production of antibodies and autoantibodies by B cells (21). On the other hand, progesterone has an immunosuppressive effect by inhibiting the proliferation of macrophages, inhibiting the secretion of Il6, and inhibiting the production of antibodies (22). Therefore, androgens show a different effect. They enhance the activity of T suppressor lymphocytes (Ts), the Th1-dependent immune response, the activation of CD8+ lymphocytes with a simultaneous reduction of natural killers (NK) cell responses and increase the production of interleukin 10 (Il10). Il10 is classified as an anti-inflammatory cytokine, which additionally inhibits the production of pro-inflammatory cytokines (23).

In a healthy woman, the immunomodulatory effect of estrogens is balanced by the action of progesterone. In women with PCOS, because of the disturbance of the proportions in the concentration of estrogens and progesterone, not only conditioned by the lack of ovulation but also the aromatization of androgens in the subcutaneous fat tissue, the immune system is over-stimulated and, consequently, the production of antibodies against the body’s own cells increases (24). On the other hand, the excess of androgens observed in women with PCOS might be protective against the development of autoimmune disease. Nevertheless, in this case, the role of estrogens as strong modulators of the immune system is essential. It seems that the balance between the concentrations of estradiol, progesterone, and testosterone plays an important role here. However, there are no data in the literature showing the influence of sex hormones on the development of PCOS in the group of pediatric patients.

In our study, we showed that in the group of girls diagnosed with both PCOS and AT, the concentration of E2 is significantly higher than in the group of girls with PCOS but without AT (p=0.04). Additionally, a significant positive correlation between AT and estradiol concentration (r_ƴ_=0.27; p=0.04) was found. Similar results were obtained by Arduc et al. (25). They reported a higher concentration of E2 in the group of PCOS patients with positive anti-TPO compared to PCOS patients with negative anti-TPO. They also showed a positive correlation between the titer of anti-TPO antibodies and the concentration of E2. However, Janssen et al. (26) obtained different results. In the group of women with PCOS and positive titer of anti-thyroid antibodies (anti-TPO or anti-TG), the concentration of E2 was insignificantly lower than that in the group with PCOS, but with negative titer of anti-thyroid antibodies. Similarly, they did not show statistically significant differences in the concentration of progesterone between the described groups.

In our study only the concentration of DHEAS was significantly increased in the group of girls with PCOS and AT (p=0.01). It is reported that DHEAS has anti-inflammatory, antioxidant, and immunoregulatory properties, which is confirmed by experimental studies conducted on mice *in vivo* and *in vitro*. The results show that DHEAS significantly increases the concentration of Th1-type cytokines interleukin 2 (Il2) and interferon alfa (IFNα) and reduces the levels of Th2-type cytokines interleukin 4 (Il4) and Il10, i.e., stimulates the Th1 immune response and suppresses the Th2 immune response by promoting a Th1/Th2 balance shift toward immunity from domination of Th1. These results provide significant evidence on the mechanism of DHEA-mediated immune function and effective protection against infectious and inflammatory reactions in animals and humans (27). Moreover, the analysis of the literature shows that the concentration of DHEAS in patients with PCOS positively correlates with better metabolic control and a lower level of inflammatory markers (8). Therefore, it is difficult to explain the reason for the higher concentration of DHEAS in the group of patients with PCOS and AT in our study. We did not state a significant difference in the concentration of T between patients with PCOS and AT and patients with PCOS who were not diagnosed with AT. Similarly, Janssen et al. (26) did not describe the differences in T concentration between the group of women with PCOS and positive titer of anti-thyroid antibodies (anti-TPO and/or anti-TG) and patients with PCOS but without positive titer of anti-thyroid antibodies. After analyzing the work of other researchers, it is reported that despite the lack of statistical significance, the concentration of T was in each case lower in the group of women with PCOS and AT compared with women with PCOS but without an additional diagnosis of AT (28–30). This may be an indirect proof of the protective effect of androgens on the development of autoimmune disease in women with PCOS.

## Conclusions

It is concluded, that high concentration of estradiol found in girls with PCOS and AT may indicate the role of this hormone in the development of the autoimmune process. Moreover, coexistence of PCOS with AT is not related to different metabolic profile comparing to adolescent girls with PCOS only. However, tendency to positive correlation between AT and the glucose concentration at 120 min OGTT and consequently higher glucose concentration in 120 min OGTT in girls with PCOS and AT may suggest an increased risk of developing type 2 diabetes in the future. It may indicate the need for early prophylaxis in the group of patients with PCOS and AT to prevent cardiometabolic complications at the period of adolescence. However, the numbers are small, and more research is needed to confirm our findings.

## Data Availability Statement

The raw data supporting the conclusions of this article will be made available by the authors, without undue reservation.

## Author Contributions

KS participated in the conception of the work, acquisition and interpretation of data for the work, drafting the work. AZ participated in revising the work for important intellectual content. AG participated in evising the work for important intellectual content. All authors contributed to the article and approved the submitted version.

## Funding

Śląski Uniwersytet Medyczny.

## Conflict of Interest

The authors declare that the research was conducted in the absence of any commercial or financial relationships that could be construed as a potential conflict of interest.

## References

[1] Fauser BC Tarlatzis RW Rebar RS Legro RS Balen AH Lobo L. Consensus on Women`s Health .Aspects of Polycystic Ovary Syndrome (PCOS): The Amsterdam Eshre/ ASRM-Sponsored 3rd PCOS Consensus Workshop Group. Fertil Steril (2012) 97:28–38.e25. 10.1016/j.fertnstert.2011.09.024 22153789

[2] Legro RS Kunselman AR Dod- son WC Dunaif A. Prevalence and Predictors of Risk for Type 2 Diabetes Mellitus and Impaired Glucose Tolerance in Polycystic Ovary Syndrome: A Procpective, Controlled Study in 254 Affected Women. J Clin Endocrinol Metab (1999) 84:165–9. 10.1210/jcem.84.1.5393 9920077

[3] Canaris GJ Manowitz NR Mayor G Ridgway EC. The Colorado Thyroid Disease Prevalence Study. Arch Intern Med (2000) 160:526–34. 10.1001/archinte.160.4.526 10695693

[4] Artini PG Uccelli A Papini F Simi G Di Berardino OM Ruggiero M. Infertility and Pregnancy Loss in Euthyroid Women With Thyroid Autoimmunity. Gynecol Endocrinol (2013) 29:36–41. 10.3109/09513590.2012.705391 22835333

[5] Tomer Y Davies TF. Searching for the Autoimmune Thyroid Disease Susceptibility Genes: From Gene Mapping to Gene Function. Endocrinol Rev (2003) 24:694–717. 10.1210/er.2002-0030 14570752

[6] Pucci` E Chiovato L Pinchera A. Thyroid and Lipid Metabolism. Int J Obes (2000) 24:109–12. 10.1038/sj.ijo.0801292 10997623

[7] Neves JS Cunha C Neves C. Cardiovascular Risk Factors in Patients With Autoimmune Thyroiditis. Endocrine Abstracts (2017) 49:199. 10.1530/endoabs.49.GP199

[8] Ho CW Chen HH Hsieh MC Chen CC Hsu SP Yip HT. Increased Risk of Polycystic Ovary Syndrome and It’s Comorbidities in Women With Autoimmune Thyroid Disease. Int J Environ Res Public Health (2020) 17:2422. 10.3390/ijerph17072422 PMC7177418 32252386

[9] Ibanez L Oberfield SE Witchel S Auchus RJ Chang RJ Codner E. An International Consortium Update: Pathophysiology, Diagnosis, and Treatment of Polycystic Ovarian Syndrome in Adolescence. Horm Res Pediatr (2017) 88:371–95. 10.1159/000479371 29156452

[10] Szczeklik A. Interna Szczeklika. Podręcznik Chorób Wewnętrznych 2017. Medycyna Praktyczna 2017. pp. 1316–7.

[11] Ehrmann DA Barnes RB Rosenfield RL Imperial J. Prevalence of Impaired Glucose Tolerance and Diabetes in Women With Polycystic Ovary Syndrome. Diabetes Care (1999) 22:141–6. 10.2337/diacare.22.1.141 10333916

[12] Wild RA Carmina E Diamanti-Kandarakis E Dokras A Escobar–Morreale HF Futterweit W. Assessment of Cardiovascular Risk and Prevention of Cardiovascular Disease in Women With the Polycystic Ovary Syndrome: A Consensus Statement by the Androgen Excess and Polycystic Ovary Syndrome (Ae-PCOS) Society. J Clin Endocrinol Metab (2010) 95:2038–49. 10.1210/jc.2009-2724 20375205

[13] Biondi B Klein I. Hypothyroidism as a Risk Factor for Cardiovascular Disease. Endocrine (2004) 24(1):1–13. 10.1385/ENDO:24:1:001 15249698

[14] Wild RA Rizzo M Clifton S Carmina E. Lipid Levels in Polycystic Ovary Syndrome: Systematic Review and Meta-Analysis. Fertil Steril (2011) 95(3):1073–9. 10.1016/j.fertnstert.2010.12.027 21247558

[15] Amiri M Tehrani FR Behboudi-Gandevani S Carmina E. Risk of Hypertension in Women With Polycystic Ovary Syndrome: A Systematic Review, Meta-Analysis and Meta-Regression. Reprod Biol Endocrinol (2020) 18:23. 10.1186/s12958-020-00576-1 32183820 PMC7076940

[16] Scicchitano P Dentamaro I Carbonara R Bulzis G Dachille A Caputo P. Cardiovascular Risk in Women With Pcos. Int J Endocrinol Metab (2012) 10(4):611–8. 10.5812/ijem.4020 PMC3693634 23843832

[17] Kim JJ Yoon JW Kim MJ Kim SM Hwang KR Choi YM. Thyroid Autoimmunity Markers in Women With Polycystic Ovary Syndrome and Controls. Hum Fertil (2020) 7:1–7. 10.1080/14647273.2019.1709668 31910041

[18] Hulman A Gujral UP Narayan KMV Pradeepa R Mohan D Anjana RM. Glucose Patterns During the OGTT and Risk of Future Diabetes in an Urban Indian Population: The CARRS Study. Diabetes Res Clin Pract (2017) 126:192–7. 10.1016/j.diabres.2017.01.009 PMC5408861 28259008

[19] Neves C Esteves C Neves JS. Cardiovascular Risk Factors in Autoimmune Thyroiditis and Subclinical Hypothyroidism. Endocrine Abstracts (2015) 37:26. 10.1530/endoabs.37.GP.26.07

[20] Toulis KA Goulis DG Farmakiotis D Georgopoulos NA Katsikis I Tarlatzis BC. Aadiponectin Levels in Women With Polycystic Ovary Syndrome: A Systematic Review and a Meta-Analysis. Hum Reprod Update (2009) 15(3):297–307. 10.1093/humupd/dmp006 19261627

[21] Penneli LM Galligan CL Fish EN. Sex Affects Immunity. J Autoimmun (2012) 38:282–91. 10.1016/j.jaut.2011.11.013 22225601

[22] Seli E Arici A. Sex Steroids and the Immune System. Immunol Allergy Clin (2002) 22:407–33. 10.1016/S0889-8561(02)00017-6

[23] Quintero OL Amador-Patarroyo MJ Montoya-Ortiz G Rojas-Villarraga A Anaya JM. Autoimmune Disease and Gender: Plausible Mechanism for the Female Predominance of Autoimmunity. J Autoimmun (2011) 38:109–19. 10.1016/j.jaut.2011.10.003 22079680

[24] Gaberscek S Zaletel K Schwetz V Pieber T Obermayer-Pietsch B Lerchbaumet E. Thyroid and Polycystic Ovary Syndrome. Eur J Endocrinol (2015) 172:9–21. 10.1530/EJE-14-0295 25422352

[25] Arduc A Dogan BA Bilmez S Nasiroglu NI Tuna MM Isik S. High Prevalence of Hashimoto’s Thyroiditid in Patients With Polycystic Ovary Syndrome: Does the Imbalance Between Estradiol and Progesterone Play a Role? Endocr Res (2015) 40(4):204–10. 10.3109/07435800.2015.1015730 25822940

[26] Janssen OE Mehlmauer N Hahn S Offner AH Gärtner R. High Prevalence of Autoimmune Thyroiditis in Patients With Polycystic Ovary Syndrome. Eur J Endocrinol (2004) 150:363–9. 10.1530/eje.0.1500363 15012623

[27] Cao J Yu L Zhao J Ma H. Effect of Dehydroepiandrosterone on the Immune Function of Mice In Vivo and In Vitro. Mol Immunol (2019) 112:283–90. 10.1016/j.molimm.2019.06.004 31228660

[28] Kachuei M Jafari F Kachuei A Keshteli AH. Prevalence of Autoimmune Thyroiditis in Patients With Polycystic Ovary Syndrome. Arch Gynceol Obstet (2012) 285:853–6. 10.1007/s00404-011-2040-5 21866332

[29] Garcia-Garcia E Vazquez-Lopez MA Garcia-Fuentes E Galera-Martínez R Gutiérrez-Repiso C García-Escobar I. Thyroid Function and Thyroid Autoimmunity in Relations to Weight Status and Cardiovascular Risk Factors in Children and Adolescents: A Population-Based Study. J Clin Res Pediatr Endocrinol (2016) 8(2):157–62. 10.4274/jcrpe.2687 PMC5096470 26761948

[30] Unnikrishnan AG Kalra S Sahay RK Bantwal G John M Tewariet N. Prevalence of Hypothyroidism in Adults: An Epidemiological Study in Eight Cities of India. Indian J Endocrinol Metab (2013) 17(4):647–52. 10.4103/2230-8210.113755 PMC3743364 23961480

